# Gastric outlet obstruction in uncomplicated mesentero-axial gastric volvulus associated to hiatal hernia

**DOI:** 10.1016/j.radcr.2024.03.023

**Published:** 2024-04-17

**Authors:** Rosita Comune, Francesco Guida, Giampaolo Marte, Domenico Diglio, Rosano Nicola, Giacomo Bonito, Michele Tonerini, Michele Galluzzo, Mariano Scaglione, Stefania Tamburrini

**Affiliations:** a Division of Radiology, Università degli Studi della Campania Luigi Vanvitelli, Naples, Italy; b Department of General and Emergency Surgery, Ospedale del Mare, ASL NA1 Centro, Naples, Italy; c Department of Radiology Hospital of Marcianise, 81025 Marcianise, Italy; d Department of Radiology, Ospedale del Mare, ASL NA1 Centro, Naples, Italy; e Department of Emergency Radiology-Policlinico Umberto I Hospital, Sapienza University of Rome, Rome, Italy; f Department of Emergency Radiology, Cisanello Hospital, Via Cisanello, Italy; g Department of Emergency Radiology, San Camillo Forlanini Hospital, Rome, Italy; h Department of Medicine, Surgery and Pharmacy, University of Sassari, Piazza Università, Sassari, Italy; i Department of Radiology, James Cook University Hospital, Middlesbrough, UK

**Keywords:** Volvulus, Gastric volvulis, Hiatal hernia, Gastric outlet obstruction

## Abstract

Gastric volvulus is a rare condition determined by the rotation of one part of stomach around another. Stomach can rotate around its longitudinal or short axis or both. The presentation can be acute, subacute and chronic due to twisting and untwisting andimaging should be performed in the acute phase. MDCT shows high accuracy in thediagnosis and definition of gastric volvulus being the preferred diagnostic test in emergency settings. Gastric volvulus may be associated or determined by pre-existing hiatal hernia and accurate analysis of CT signs may be evaluated on order to differentiate between a stomach in an abnormal position and a volvulus.At CT, a displaced antrum at the same level or cranial to the fundus and a transition point at the pylorus is diagnostic for mesenteroaxial volvulus. We present a case of a 70 years old woman with mesenteroaxial volvulus in hiatal hernia.

## Introduction

Gastric volvulus is defined as an abnormal degree of rotation of 1 part of the stomach around another [1] and it was first described by Berti in 1866. It is a rare condition recurrent in adult population after the fifth decade of life [2]. Gastric volvulus can be complete or incomplete depending on the grade of rotation. Closed loop obstruction and strangulation may occur with consequent ischemic necrosis and perforation when the rotation exceeds 180° [2], [3], [4]. Gastric volvulus can be idiopathic due to laxity of gastric stabilizing ligament in 30% of patients, or secondary to paraoesophageal hernia, diaphragmatic eventration or paralysis, adhesions, neoplasm, or trauma [5,6]. Gastric volvulus may present as an acute abdominal emergency or as a chronic cause of upper abdominal discomfort. Subacute or intermittent presentation is due to episodic twisting and untwisting. Borchardt's triad is believed to be clinically diagnostic for acute gastric volvulus and consists of unproductive retching, epigastric pain, and distention, and the inability to pass a nasogastric tube. This triad was defined by Carter et al. prior the introduction of the flexible gastroscopy and CT [7]. Symptom may be nonspecific and mortality from acute gastric volvulus has been reported to be as high as 30%-50% [8]. Diagnostic imaging is useful when performed in the symptomatic interval instead imaging studies performed in the well interval may be nondiagnostic [5,6,9]. There are 3 anatomic types of gastric volvulus: organoaxial, mesenteroaxial, and a combination of both. The knowledge of gastric anatomy and axis are the cornerstone of the diagnosis [2]. We present a case of symptomatic uncomplicated mesenteroaxial gastric volvulus in hiatal hernia.

## Case presentation

**Fig. 1:**
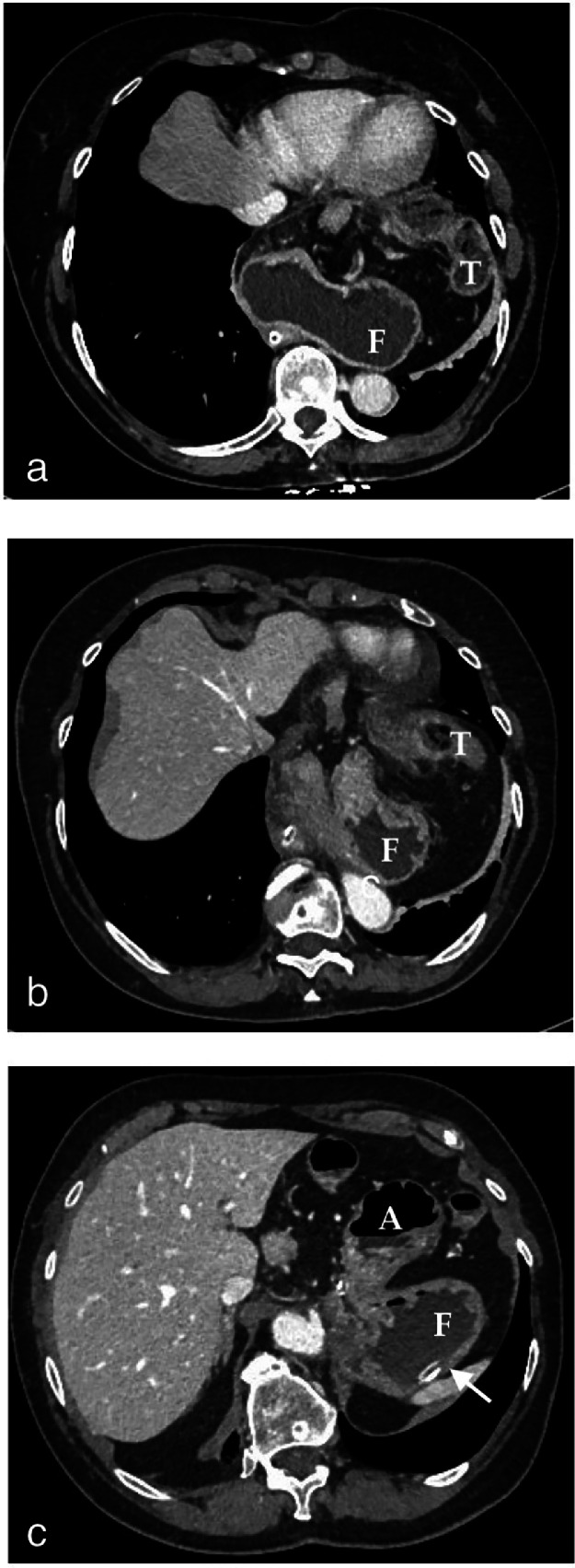
(A-C) Axial contrast enhanced CT with intravenous contrast showing a left large hiatal hernia. The transverse colon (T) is also detected within the hernial sac. The stomach appeared folded on itself with the antrum (A) at the same level and over the fundus(F). Nasogastric tube is visible at the fundus (white arrow).

**Fig. 2:**
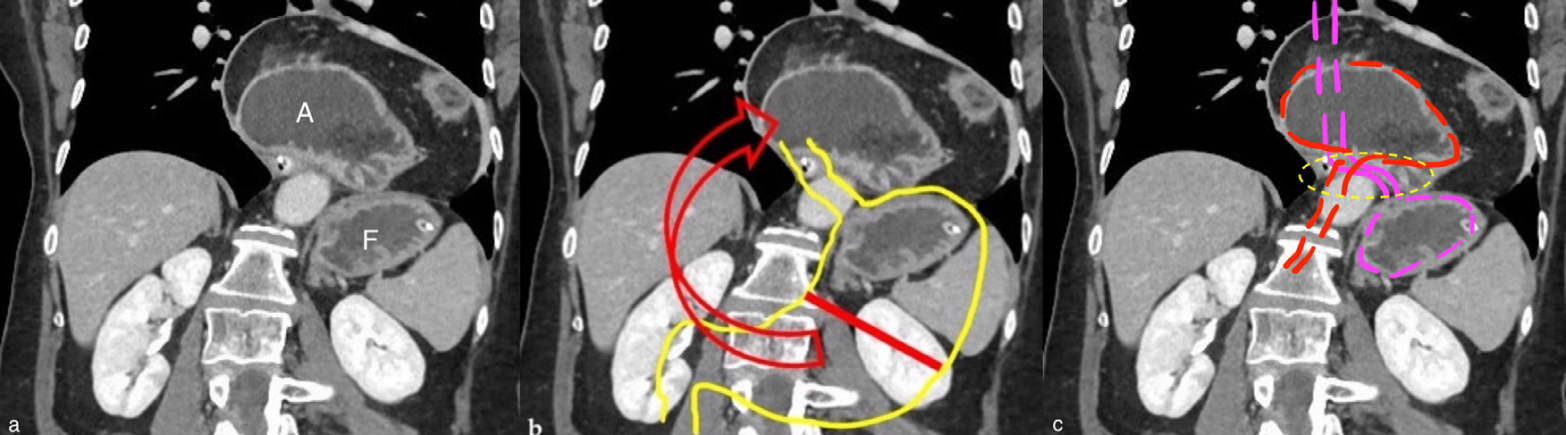
(A-C) Coronal MRP CT reconstruction. (A) Hiatal hernia with left elevated diaphragmatic dome. Gastric fundus was detected below diaphragmatic dome, antrum is displaced superiorly into the hernial sac in the thorax. (B) Schematic draw of normal anatomy (yellow line), gastric short axis (red line). Arrow indicates the displacement of the stomach in the upper direction and rotation around the short axis from the minor to the major curvature. (C) Schematic draw of mesenteroaxial volvulus that shows the cardias and fundus (purple dashed line) below the left diaphragm and the displaced body and antropiloric junction (red dashed line) with. The yellow oval dashed circle indicates the collar neck of the hiatal hernia.

**Fig. 3:**
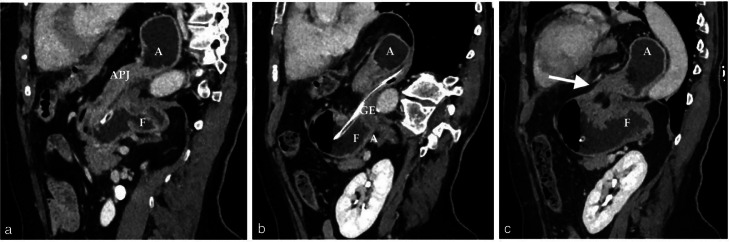
(A-C) Sagittal MRP CT reconstruction. Gastric fundus (F) is located below the left hemidiaphragm, the antrum (A) is displaced cranially into the thorax. The gastroesophageal junction with nasogastric tube (GE) lies posteriorly. The antropyloric junction (APJ) is displaced anteriorly. Gastric stenosis is clearly visible at the hernial neck (white arrow).

**Fig. 4:**
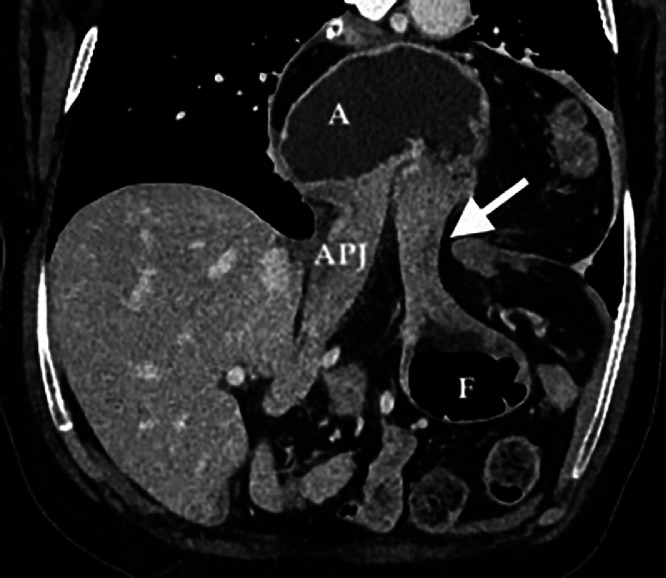
Paracoronal MPR reconstruction clearly demonstrated the twisting and herniation of gastric body into the hiatal hernia. Hernial neck and transition point at antropyloric junction is evident (white arrow).

We report a case of a 70-year-old woman who complained of 1 and half year's history of intermittent epigastric pain. Because of the intermittent epigastric pain, she previously underwent cardiological exams with unremarkable results. She was diagnosed with hiatal hernia of unknown type and treated for esophageal reflux. She had no history of abdominal surgeries. She presented at ER for acute epigastric pain with hematemesis, unproductive retching, and abdominal distension. Physical examination revealed abdominal distension and mild epigastric pain without peritonism. Cardiorespiratory auscultation was normal. She was afebrile and hemodynamically stable. Complete blood count and metabolic panel were unremarkable except for white blood cells (1449 10^3^/mm^3^, n.v. 4.2-10.5) neutrophils (81.8, n.v 40,0-75) and a slightly increase of PCR (0.78 mg/dl, n.v.0.0-0.5 mg/dL). A nasogastric tube was placed difficulty. Because of the characteristic symptomatology, the history of hiatal hernia, and the obstacle encountered to complete insertion of nasogastric tube, a suspected diagnosis of gastric outlet obstruction was formulated. The patient underwent MDCT with intravenous contrast (1.0-1.5 mL/kg injected at 3.5 mL/s, followed by 50 mL of a saline bolus injection). A CT multiphase protocol was acquired (noncontrast, arterial, and parenchymal). At CT, a large hiatal hernia with elevated left hemidiaphragm was appreciated. Transverse colon also was herniated and displaced superiorly in the hernial sac (Figs. 1A-C). The nasogastric tube was in the gastric fundus placed below the diaphragm. The stomach appeared folded on itself with the antrum located superiorly over the fundus. The antropyloric junction (APJ) was displaced superior and anterior to gastroesophageal junction (GEJ) (Figs. 2A-C). A transition point at the pylorus and stenosis at the hernia neck were detected (Figs. 3A and 4). These aspects reflected a stomach torsion around its short axis due to mesenteroaxial volvulus into a hiatal hernia.

Because of the absence of signs of parietal vascular damage, the patient was admitted to the hospital and underwent gastroscopy. At EGD (esophagogstroduodenoscopy), the cardias appeared high in position and incontinent, an erythematous Z-line, coincident with the gastric esophageal junction, was positioned at 38 cm from the upper dental arch. At cardia site, a small ulceration with fibrine was detected probably caused by unproductive retching. Fundus and body appear rotated and “likely” ascending into the thorax.

The patient underwent robotic surgery (Da Vinci Robot -Intuitive Surgical Inc., Sunnyvale, CA) under general anesthesia, the patient was placed in reverse Trendelenburg.

The gastro-hepatic ligament was sectioned and the hernia sac at the level of the right crus level allowing successful dissection of the sac and the reduction of hernia in the abdomen. After the identification of the anterior vagus nerve, the stomach was positioned into the abdomen difficulty due to the presence of multiple adhesions. A wide retroesophageal window was created and a penrose drain was placed to retract the distal esophagus.

The diaphragmatic crura was closed with 3 1-cm distance non absorbable stitches of 2-0 silk.

An “on-demand” separation of short gastric vessels was performed to reduce the chance for postoperative dysphagia and gas bloating. The bougie was positioned into the stomach and the fundoplication was created. A 3-cm length “floppy” fundoplication was performed with two 2-0 silk stitch that closed the 2 flaps of the fundus without incorporating the esophageal wall. After completing the fundoplication, intraoperative upper endoscopy was performed.

One drainage was placed proximal to fundoplication and of crura suture. Postoperatively, the patient was stable and with good recovery.

## Discussion

Gastric volvulus is usually divided into 3 subtypes: organoaxial, mesenteroaxial, or both. Organoaxial volvulus is more common than mesenteroaxial volvulus and accounts for about two-thirds of cases. In organoaxial volvulus, the stomach rotates along its longitudinal axis that is represented by the axis that connects both gastric outlets along the cardiopyloric line [5]. In these cases, the greater curvature is displaced superiorly the lesser curvature is located inferiorly, fundus posteroinferiorly, and antrum anterosuperiorly [5,10]. In organoaxial volvulus the stomach appears a mirror images of its normal anatomy [5]. Organoaxial volvulus most commonly occurs in the setting of trauma or paraesophageal hernia [4,10]. In mesenteroaxial volvulus, the stomach rotates along its short axis, with the pylorus flipping superiorly. The axis of twisting causes the antrum and pylorus to end up cranially relative to both the proximal body and the fundus. Stomach short axis is perpendicular (90 degrees) to the cardiopyloric line and connects lesser and greater curvatures [5]. In these cases, the antrum is displaced above the gastroesophageal junction, proximal body, and fundus. In mixed volvulus the rotation occurs along both longitudinal and axial axis [5,11,12]. Radiographic findings of gastric volvulus include a spherical stomach or a double air-fluid level on upright chest films, a retrocardiac air-fluid level above an elevated left diaphragm dome and collapsed small intestine. Nasogastric tube if in place may appear abnormally in the chest in organoaxial volvolus [13]. A barium swallow and upper GI endoscopy may be helpful as an adjunct to chest X-ray, but they are time-consuming in emergency setting, and have been replaced by contrast CT [14]. EGD can be diagnostic in less than 50% of cases [15], it can reveal a tortuous appearance of the stomach and difficulty or inability to reach the pylorus [16]. Endoscopic reduction has been reported but does not address the underlying pathology that predisposes to torsion of the stomach [17]. MDCT with intravenous contrast is highly sensitive and specific for the diagnosis of gastric volvulus with an overall accuracy of 90% [6,10,18] providing a comprehensive description of the thoracic lesion, including stomach vitality [19]. CT diagnosis of gastric volvulus can be difficult, for these reason radiologists should be familiar with useful CT findings to achieve a prompt an early diagnosis [6,20,21]. The difficulty in the diagnosis of gastric volvulus is that it frequently occurs in the settings of large hiatal or diaphragmatic hernia: hiatal hernia and gastric volvulus have similar appearance with abnormal positing of the stomach [5,6,20,22], so other signs should be carefully reviewed. Multiplanar CT images and CT3D angiographic reconstruction may be helpful in order to accurately determine the anatomy and the degree of rotation and to follow the course of the right gastric vein dorsally across the stomach and up along what would be normally be the major curvature [2]. Few CT findings have been described in CT diagnosis of gastric volvulus. Millet et al found that the combination of the antrum at the same level or cranial to the fundus and a transition point at the pylorus have 100% sensitivity and specificity for the diagnosis of gastric volvulus [20]. Marahemi et al. reported that the transition point at the pylorus and stenosis at the hernia neck are the most frequent findings with high sensitivity and specificity and interobserver agreement, also allowing the differential diagnosis with hiatal hernias [6]. The transition point at the pylorus is relatively easy to identify and it is extremely useful, in common practice, instead stenosis at the hernia neck may be not clear in large hiatal hernias. On the other hand, the absence of these 2 signs decreases the posttest probability of having gastric volvulus [6]. In complicate gastric volvulus, CT signs of ischemia such as parietal edema or hypoenhancement, perigastric fluid, pneumoperitoneum, and associated pleural effusion increase the diagnostic confidence of gastric volvulus. Gastric volvulus has been also reported in associate to congenital or acquired splenic hypermobility [23].

Gastric outlet obstruction refers to a mechanical obstruction of the pylorus or the duodenum that may be cause by benign or malignant diseases [24]. In our case it was determined a mesenteroaxial gastric volvulus in hiatal hernia in an old woman, which was rare because it is more commonly reported in neonates, infants, and young children [25,26]. Clinical symptoms and previously history of hiatal hernia was suggestive of mechanical gastric obstruction and the patients was imaged with MDCT with intravenous contrast. CT scans provided an accurate diagnosis with specific anatomical details assessing also the absence of parietal damage and the herniation of the transverse colon. Position of the antrum at the same level to the fundus, a transition point at the pylorus and stenosis at the hernia neck allowed a confident CT diagnosis of mesenteroaxial volvulus. EGDS performed during the hospitalization confirmed the ascending position of gastric corpus. The definitive treatment was surgical allowing the repair of the volvulus and decreases the chance of recurrence through diaphragmatic hernia correction.

## Conclusion

In gastric volvulus both clinical exams, EGD and radiography can be indeterminate, leading to CT being the preferred modality. CT diagnosis of gastric volvulus may be challenging because it frequently occurs in hiatal hernia, and it may be difficult to differentiate the abnormal position of the stomach. Knowledge of stomach anatomy and axis is fundamental to recognize gastric rotation. Because gastric volvulus can present in acute, subacute or intermittent form, it is important to evaluate the patient in the symptomatic phase to avoid false negative diagnosis. A displaced antrum at the same level or cranial to the fundus and a transition point at the pylorus are highly suggestive of mesenteroaxial gastric volvulus.

## Patient consent

Written informed consent was obtained from the patient for publication of this case report and accompanying images. A copy of the written consent is available for review by the Editor-in-Chief of this journal on request.
